# Correlation of Abdominal Fat Distribution with Different Types of Diabetes in a Chinese Population

**DOI:** 10.1155/2013/651462

**Published:** 2013-11-10

**Authors:** Anhui Zhu, Bin Cui, Haodan Dang, Dan Yao, Haitao Yu, Hongmin Jia, Zhijun Hu, Xiaojin Zhang

**Affiliations:** Department of Radiology, Aerospace Central Hospital, 15 Yu'quan Road, Haidian District, Beijing 100049, China

## Abstract

To investigate abdominal fat distribution in Chinese subjects with diabetes and its correlation with different types of diabetes. A total of 176 diabetic subjects were enrolled, 92 with type 1 and 84 with type 2, with a mean age of 27.41 and 49.3 yrs. No subject has history of severe diseases. Multi-slice CT was used to measure total abdominal adipose (TA) and visceral adipose (VA) tissues. Subcutaneous adipose (SA) tissue was obtained by subtracting VA from TA. There were differences between subjects with T1DM and T2DM for TA, VA, SA, VA/SA, body mass index (BMI), triglyceride (TG) and high density lipoprotein, but not total Cholesterol or low density lipoprotein. There were positive correlations between TA, VA, SA, VA/SA and T1DM and T2DM (*P* < 0.05 and *r* > 0.86). In subjects with T1DM, VA was negatively correlated with HDL, positively with BMI and age, and SA was positively correlated with BMI and sex (*P* < 0.05 and *r* > 0.86 for all). In subjects with T2DM, VA was positively correlated to BMI, TG and age, and SA was positively correlated to TG and sex (*P* < 0.05 and *r* > 0.86 for all). Abdominal fat content was positively correlated to diabetes in Chinese, which differs in different types of diabetes.

## 1. Introduction

Glucose and lipid metabolism are closely interacted with each other. When lipid metabolism is in disorder, glucose metabolism would be interfered. On the other hand, fat is deposited in different regions of the body, and people become obese. It is well known that obesity is highly associated with diabetes. There are many methods to evaluate obesity, such as BMI and waist circumference (WC) [1, 2]. Although these parameters are helpful for diagnosis of obesity, they do not demonstrate the correlation of obesity and other diseases [3]. With the development of medical imaging techniques, there are more and more techniques used for measuring body fat distribution in human being, such as CT and magnetic resolution imaging (MRI). Especially MSCT, whose exact value can be used to determine the nature of tissues, combined with the process of imaging, can measure the area of fat precisely, with minor error. MSCT has been regarded as the gold standard for measuring fat distribution [4–6].

There was a study that compared abdominal fat distribution in different types of diabetes and their correlations [7]. However, it is done for different regions of the body such as fat in skeletal muscle and thigh, and it was performed in Canadians. Fat distribution varied in different ethnic groups [8] and thus had different contribution to the onset of diabetes [9]. As per our knowledge, there is no study investigating the abdominal fat distribution in different types of diabetes or its contributions in Chinese population.

Therefore, in this study, by using MSCT, we measured the abdominal fat of Chinese subjects with diabete, and explored the characteristics of abdominal fat distribution in Chinese subjects with different types of diabetes, as well as its correlation with diabetes in Chinese population.

## 2. Materials and Methods

### 2.1. Study Population

This study was approved by ethics board and institutional review board of our hospital. All the subjects signed the written consent form prior to the study initiation. All the procedures meet the requirement of clinical research and health insurance portability and accountability act compliance. From those who visited our department during Jan of 2010 to Jan of 2013, 176 subjects with diabetes were randomly enrolled into this study, including 92 with T1DM and 84 with T2DM. There were 51 males and 41 females in T1DM group, aged 14~59 yrs, with a mean age of 27.41 ± 11.03 yrs. There were 60 males and 24 females in T2DM group, aged 23~64 yrs, with a mean age of 49.3 ± 10.11 yrs. Total abdominal adipose tissue (TA) and visceral adipose tissue (VA) were measured with MSCT and histogram method. Subcutaneous adipose tissue (SA) area was obtained by subtracting VA from TA. The ratio of visceral adipose tissue and subcutaneous adipose tissue (VA/SA) was calculated. Other characteristics such as BMI, TG, HDL, total cholesterol (Tch), and LDL were collected in the whole population enrolled. All the measurements were performed per manufacturer protocol.

### 2.2. Methods

#### 2.2.1. CT Scanning

GE LightSpeed 16 spiral CT was used. Scan was performed at the umbilicus level (L4), with the axial view, a voltage of 120 Kv, a current of 200 mA, and by Axial/2i, for 5 mm of thickness and an interval of 5 mm. The imaging was rebuilt in stand mode.

#### 2.2.2. Measurement of Abdominal Adipose Content

**Figure 1:**
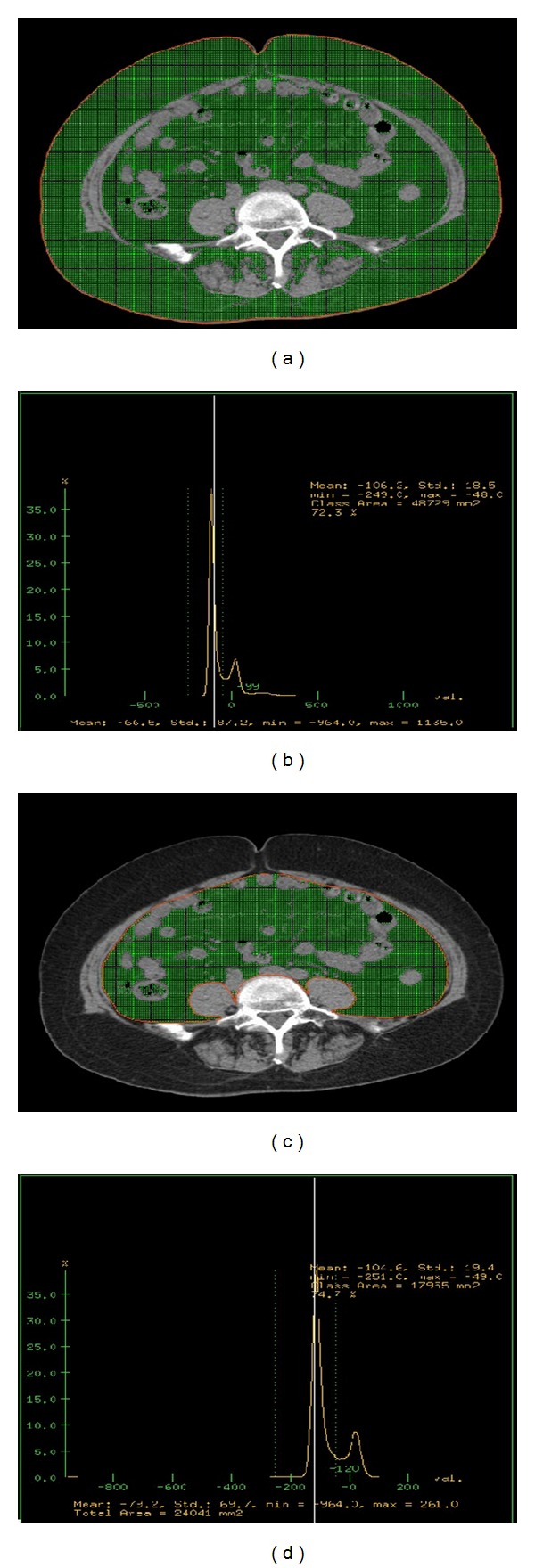
(a)–(d) are the measurement for abdominal fat. (a) is the line along abdominal skin at the axial position of CT image (red line); (b) is the area within adipose tissue of this area with histogram calculation (green area), that is, the total abdominal adipose tissue (TA) (mm^2^); (c) is the line along the abdominal wall and the internal edges of bilateral psoas major (red line); (d) is the area of adipose tissue within this area with histogram calculation (green area), the visceral adipose tissue (VA) (mm^2^).

Transfer the imaging to ADW4.4 workstation for process, set the CT value of adipose tissue attenuating region as −50~−250 Hu with histogram method, and draw a line along the abdominal skin. Within this area of adipose tissue, the total abdominal adipose tissue (TA) (mm^2^) (Figures 1(a) and 1(b)) was measured. Draw another line along the abdominal muscles and the internal edges of bilateral psoas, and measure adipose content within this area, which is the visceral adipose tissue area (VA) (mm^2^) (Figures 1(c) and 1(d)). The difference between TA and VA was then calculated, by which the subcutaneous adipose area (SA) (mm^2^) was obtained. The ratio of VA/SA was then calculated. The measurements were conducted by 2 experienced CT who were radiologists blinded to subjects' information. Data was then reviewed by the third radiologist. The mean of the measurements was used as the final value for analysis.

#### 2.2.3. Statistical Analysis

SPSS13.0 was used for data analysis. Data consistency between two measurements by two radiologists was tested with *Kapper* test. The basic parameters among subjects with different types of diabetes were analyzed with student *t*-test. The correlations were performed with linear regression analysis (stepwise). The data were presented as mean ± SD. *P* < 0.05 was regarded significant.

## 3. Results

### 3.1. Data Consistency by Two Radiologists Was Tested with Kapper Test

The *Kapper* value for these two radiologists was >0.8, suggesting a good consistency between radiologists. The mean of these two measurements was used for analysis.

### 3.2. Characteristics of Abdominal Fat Distribution in Subjects with Different Types of Diabetes

As shown in Table 1, there were significant differences for TA, VA, SA, VA/SA, BMI, TG, and HDL between subjects with T1DM and those with T2DM (*P* < 0.05), all but HDL higher in T2DM, but not for Tch or LDL.

### 3.3. Correlation Analysis for Abdominal Fat Content with Different Types of Diabetes

#### 3.3.1. Correlation Analysis for Abdominal Fat Content with Type 1 Diabetes

As shown in Table 2, TA, VA, SA, and VA/SA are positively correlated to the onset and duration of T1DM (*P* < 0.05), and the correlation coefficients of TA and SA were relatively higher. According to the correlation equation, TA and VA were negatively correlated with HDL, TA was positively correlated with BMI, AGE, and SEX, VA was positively correlated with BMI and AGE, and SA was positively correlated with BMI and SEX (*P* < 0.05).

#### 3.3.2. Correlation Analysis for Abdominal Fat Content with Type 2 Diabetes

TA, VA, SA, and VA/SA are positively correlated to the onset of type 2 diabetes, and the correlation coefficient with SA was relatively high. According to the correlation equation, TA and VA were positively correlated with TG or SEX, and VA was positively correlated with BMI, AGE, and TG (Table 3).

## 4. Discussion

Currently, fat distribution has been widely used for the studies in metabolic disease in many institutes. It is confirmed that the bigger the area with subcutaneous and visceral adipose tissue, the higher the incidence of related diseases, such as intolerant glucose test, diabetes mellitus, insulin resistance, and lipids metabolic disorders [10–17]. In this study, it is found that TA, VA, SA, and VA/SA are positively correlated to both type 1 and type 2 diabetes. The subjects with VA/SA >1 account for 40.48% (34/84) of those with type 2 diabetes, whereas the number is only 2.18% (2/92) for subjects with type 1 diabetes, statistically significant. This suggested that compared with subjects with type 2 diabetes, the subjects with type 1 diabetes have more subcutaneous fat than visceral fat. This seems not to be reported previously and may be a novel finding.

Wagenknecht et al. [10] showed that abdominal fat distribution is closely associated with insulin resistance and insulin sensitivity. Hayashi et al. [15] revealed that abdominal fat distribution was significantly correlated with glucose tolerance abnormality. There were very few studies in Chinese population. It is shown [3] that abdominal fat content is positively correlated to insulin resistance. While Li et al. [18] demonstrated that abdominal fat is an independent factor for insulin sensitivity. However, these studies were for subjects with type 2 diabetes. So far, there is no report for subjects with T1DM. Furthermore, most the subjects in those studies were middle aged or the elders. There is no large study for younger aged. In this study, the mean age of the subjects was below 60 yrs old. With the comparison between T1DM and T2DM, it is shown that there were significant differences for TA, VA, SA, VA/SA, BMI, TG, and HDL between T1DM and T2DM in a Chinese population. The levels of TA, VA, SA, VA/SA, BMI, and TG were higher in subjects with T2DM than those with T1DM, but lower for HDL. Furthermore, there is no difference for Tch or LDL between T1DM and T2DM, both close to normal or only slightly higher than the normal range. This may be due to the different state of obesity. In this study, the subjects with type 1 diabetes had a BMI of 21.01 ± 3.6, while that for subjects with type 2 diabetes was 25.42 ± 3.47, statistically significant. In another word, the subjects with T2DM were fatter than those with T1DM. It was reported that obesity was closely associated with T2DM and lipids disorders. Moreover, due to insulin resistance in type 2 diabetes, there is significant lipid disorders, presented as elevated TG and decreased HDL [19].

It was confirmed in [17] that abdominal fat distribution was correlated with the elder subjects with type 2 diabetes (≥65 yrs). There is no report in subjects with T1DM. In this study, we showed that TA and VA were positively correlated to BMI and negatively with HDL in subjects with T1DM. TA was positively correlated to TG, while VA was positively correlated to BMI, TG, and age in subjects with T2DM. It can be assumed that the correlated factors of abdominal fat content in different types of diabetes are different. This may guide the individual treatment strategy in the clinic.

Another unique aspect of this study is that it is done with Chinese population. Compared with other studies done with other population [7, 20, 21], there was unique finding from our study. First, it is found that more subcutaneous fat in subjects with T1DM, and this is correlated to the state of diabetes. Secondly, different types of diabetes had different factors impacting the total abdominal fat and visceral fat.

With the development of radiology, we now can measure abdominal fat distribution with MRI and CT in human being. MRI has no radiation and has a high resolution for tissues; thus it determine the abdominal fat accumulation very well. However, it takes longer time to perform and is complicated and difficult to be used widely. On the other hand, multislice spiral CT (MSCT) is convenient and rapid [22]. It is now regarded as the gold standard to measure fat distribution. There were limitations, such as the computer software which has to be matched up, there is radiation, and it is limited for some specific populations. Nonetheless, its benefit weight over its limitations for fat measurement [23]. Another drawback of our study was that we did not compare with the normal controls for each group, as we intended to take a look at the difference between T1DM and T2DM. This will be included in our ongoing study, and the data will possibly be reported in our next paper.

In summary, MSCT is a simple, convenient, and reliable method to measure abdominal adipose tissue in human being. Per the best of our knowledge, this is the first study investigating the correlation of T1DM and abdominal fat distribution. It is also the first study evaluating the difference of abdominal fat content between T1DM and T2DM in Chinese population. It is suggested that abdominal fat content is positively correlated to the development of T1DM and T2DM in Chinese. Subcutaneous fat seems to play an important role in the onset of T1DM. Additionally, we showed that the impact factors affecting abdominal fat content in different types of diabetes are different. This may become a potential guide for individualized medicine in the clinic.

## Figures and Tables

**Table 1:** Comparison of parameters in subjects with type 1 and type 2 diabetes.

	Age	TA	VA	SA	VA/SA	BMI	TG	Tch	HDL	LDL
T1DM	27.4 ± 11.0	12562 ± 7557	3246 ± 2280	9315 ± 5905	0.41 ± 0.25	21.0 ± 3.6	0.830 ± 0.435	4.48 ± 1.21	1.2 ± 0.30	2.6 ± 0.9
T2DM	49.3 ± 10.1	34722 ± 34799	13767 ± 6711	20955 ± 33686	0.93 ± 0.46	25.4 ± 3.5	1.993 ± 1.329	4.63 ± 0.91	0.9 ± 0.23	2.8 ± 0.8
*T* value	−13.7	−5.96	−14.17	−3.26	−9.31	−8.3	−7.94	−0.90	7.44	−1.7
*P* value	<0.001	<0.001	<0.001	0.001	<0.001	<0.001	<0.001	0.369	<0.001	0.1
95% CI	−25.0	−29501	−11986	−18685	−0.623	−5.5	−1.453	−0.461	0.218	−0.5
	−18.7	−14818	−9055	−4593	−0.405	−3.4	−0.874	0.172	0.376	0.04

T1DM: type 1 diabetes mellitus; T2DM: type 2 diabetes mellitus; TA: total adipose; VA: visceral adipose; SA: subcutaneous adipose; BMI: body mass index; TG: triglyceride; Tch: total cholesterol; HDL: high density lipoprotein; LDL: low density lipoprotein.

**Table 2:** Correlation of abdominal fat in patients with type 1 diabetes.

	TA	VA	SA	VA/SA
Correlation equation	−9201.700 +970.632BMI +6689.191SEX +138.576AGE −4563.910HDL	−3492.221 +291.358BMI +79.431AGE −1299.145HDL	−9674.451 +5735.256SEX +779.121BMI	0.411 −0.249SEX +0.004AGE
*R* Square	0.551	0.489	0.512	0.299
*F* value	26.646	28.051	46.622	18.963
*P* value	<0.001	<0.001	<0.001	<0.001

**Table 3:** Correlation of abdominal fat in subjects with type 2 diabetes.

	TA	VA	SA	VA/SA
Correlation equation	11907.740 +8451.085TG +20055.980SEX	−20541.600 +1017.048BMI +133.967AGE +924.560TG	1003.820 +22210.083SEX +6693.090TG	0.544 −0.449SEX +0.010AGE
*R* Square	0.174	0.398	0.161	0.221
*F* value	8.539	17.616	7.794	11.478
*P* value	<0.001	<0.001	0.001	<0.001

TA: total adipose; VA: visceral adipose; SA: subcutaneous adipose.
